# Machine Learning Approach to Automated Quality Identification of Human Induced Pluripotent Stem Cell Colony Images

**DOI:** 10.1155/2016/3091039

**Published:** 2016-07-14

**Authors:** Henry Joutsijoki, Markus Haponen, Jyrki Rasku, Katriina Aalto-Setälä, Martti Juhola

**Affiliations:** ^1^School of Information Sciences, University of Tampere, Kanslerinrinne 1, FI-33014 Tampere, Finland; ^2^BioMediTech, University of Tampere, Biokatu 12, FI-33520 Tampere, Finland; ^3^School of Medicine, University of Tampere, Biokatu 12, FI-33520 Tampere, Finland

## Abstract

The focus of this research is on automated identification of the quality of human induced pluripotent stem cell (iPSC) colony images. iPS cell technology is a contemporary method by which the patient's cells are reprogrammed back to stem cells and are differentiated to any cell type wanted. iPS cell technology will be used in future to patient specific drug screening, disease modeling, and tissue repairing, for instance. However, there are technical challenges before iPS cell technology can be used in practice and one of them is quality control of growing iPSC colonies which is currently done manually but is unfeasible solution in large-scale cultures. The monitoring problem returns to image analysis and classification problem. In this paper, we tackle this problem using machine learning methods such as multiclass Support Vector Machines and several baseline methods together with Scaled Invariant Feature Transformation based features. We perform over 80 test arrangements and do a thorough parameter value search. The best accuracy (62.4%) for classification was obtained by using a *k*-NN classifier showing improved accuracy compared to earlier studies.

## 1. Introduction

Regenerative medicine is living a new era. Around ten years ago, Takahashi and Yamanaka demonstrated in [1] that mouse embryonic and adult fibroblasts can be reprogrammed back to stem cells by introducing four genes encoding transcription factors (Oct3/4, Sox2, Klf4, and c-MYC [2]) [3]. Obtained stem cells were called induced pluripotent stem cells (iPSCs). One year later in an article by Takahashi et al. [4], it was shown that corresponding process can be repeated with human fibroblasts and the stem cells were called, respectively, human induced pluripotent stem cells (hiPSCs).

iPS cell technology has huge potential as Yamanaka has stated [3]. This fact is true not only because of the nature of iPSCs that they can be differentiated to any cell type wanted such as functional cardiomyocytes [5, 6] but also because they remove two major problems present with embryonic stem cells [3]:

1. Immune rejection after transplantation.
2. Severe ethical questions.

Although iPS cell technology includes a lot of possibilities, there are still technical and biomedical challenges to be overcome such as teratoma formation and the uncertainty of nuclear reprogramming completeness of iPS cell clones before iPSC technology can be used, for example, for tissue repairing, disease modeling, and drug screening [3]. The use of iPSCs in disease modeling and drug screening is the most probable application to be achieved in the near future. Since derivation of iPSCs uses patient's own cells, they are genetically identical and include all possible gene mutations of specific disease or condition which enables patient specific disease modeling and drug therapy [7].

Before we can apply human iPSCs as a standard method, there are still two major problems from the computational point of view which need to be solved. Currently, the quality monitoring of growing iPSC colonies is made manually. However, in the future when iPS cell colonies are grown in large scale, the quality controlling is impossible to carry out merely by human resources. Furthermore, only iPSC colonies of good quality should be identified and used in any further applications. Hence, the quality control process must be automated by taking, for example, an image with regular intervals from the colonies. This also gives an objective perspective to the decision-making.

The problem related to quality control returns to image analysis and classification tasks which can be divided into two separate questions:

1. In the reprogramming stage by using computational methods to identify when the patient's somatic cells have been fully reprogrammed to iPSCs.
2. In the culturing stage to identify the quality of growing iPSC colonies in order to exclude the possible abnormal iPSC colonies which cannot be used in further measures.

The first challenge was tackled in a recent article by Tokunaga et al. [8] where computational methods were examined regarding how to separate somatic cells and non-iPSCs from the iPSCs. In the article wndchrm [9, 10], a multipurpose image classifier was applied to the image analysis and classification problems. However, the interest and the focus of this research are on the second challenge which can be divided into two different culture settings:

1. Automated quality identification of iPSC colony images where feeder cells are not included, that is, feeder-free system.
2. Automated quality identification of iPSC colony images where feeder cells are included.

Automated quality identification of stem cell colony images has been made also before. Jeffreys [11] used SVMs and textural-based approach to classification. Nevertheless, the stem cells were not iPSCs since the publication was published before 2006. Furthermore, in an earlier report [12], stem cell classification was investigated using texture descriptors.

In our previous study [13], the feeder-free system was examined whereas in the following reports [14, 15] the more challenging system with feeder has been analyzed. A common factor for our previous studies with feeders [14, 15] was that intensity histograms were used as a feature. In [14], baseline classification methods and one Directed Acyclic Graph Support Vector Machines (DAGSVM) [16] structure were used whereas in [15] the focus was on modified DAGSVM classification. In this current study, we address setting with feeders and compared to our previous researches [14, 15] we have included several new approaches to this paper including

1. larger image dataset,
2. new feature extraction method and a simple way to handle the features,
3. new classification methods.

More specifically, we use Scaled Invariant Feature Transformation (SIFT) [5, 17–20] instead of intensity histograms [14, 15, 21] and present a simple way to handle SIFT descriptors in classification problems. We perform over 80 test setups and thorough parameter value search. From the baseline classification methods, we use *k*-nearest neighbor (*k*-NN) classifier [14, 22–26] with different distance measures and distance weightings. Moreover, classification tree [25, 27, 28], linear discriminant analysis [23, 29–31], multinomial logistic regression [32, 33], and naïve Bayes [23, 28, 29, 34] with and without kernel smoothing density estimation [29] were used. Quadratic discriminant analysis [23] and discriminant analysis using Mahalanobis distance [35] were tested but these could not be evaluated due to nonpositively definite covariance matrix. From multiclass SVMs, we tested DAGSVM [16, 36–40], one-versus-all (OVA) [36, 37, 41–43], one-versus-one (OVO) [36, 37, 41, 42, 44], and binary tree SVM [45, 46]. We repeated our experimental SVM tests with seven kernels and in binary tree SVMs and DAGSVM we tested all possible orders what can be constructed from our classes (good/semigood/bad) in a dataset. Furthermore, we used Least-Squares SVM [47–51] in all our tests.

The paper has the following structure. In Section 2, we describe briefly the theory of binary Least-Squares Support Vector Machine classifier and give a presentation of the multiclass extensions used. Since the baseline classification methods are well known, a reader can find the descriptions of the methods from the aforementioned references.  Section 3 presents the data acquisition procedure as well as the description of image dataset. A thorough description of design of experiments from the feature extraction and classification procedure are given in Section 4, and Section 5 is for the results and their analysis. Finally, Section 6 is for discussion and conclusion.

## 2. Methods

### 2.1. Least-Squares Support Vector Machines

Least-Squares Support Vector Machine (LS-SVM) is a reformulation from traditional Vapnik's SVM [52–54] which is solved by means of quadratic programming. LS-SVM was developed by Suykens and Vandewalle [48–50]. The starting point for derivation of LS-SVM classifier is that we have a training set {(**x**_*i*_, *y*_*i*_)}_*i*=1_^*m*^, where **x**_*i*_ ∈ *ℝ* ^*n*^ and *y*_*i*_ ∈ {−1,1}. According to [15, 48–50], LS-SVM optimization problem can be presented as follows:

(1) minw,b,e JPw,e=12wTw+C2∑i=1mei2,

where inequality constraints (when comparing to Vapnik's SVM) have been changed to equality constraints:

(2) $$\begin{array}{c} y_{i}\left[\;w^{T}\phi \left(\;x_{i}\;\right)+b\;\right]=1-e_{i},\;i=1,2,\ldots ,m, \end{array}$$

where *e*_*i*_ is an error variable for the *i*th training example and *ϕ*(·) is a nonlinear mapping into a higher dimensional feature space. The Lagrangian for the aforementioned optimization problem is now based on [15, 48–50]

(3) Lw,b,e,α=JPw,e−∑i=1mαiyiwTϕxi+b−1+ei,

where Lagrangian multipliers can have positive and negative values due to (2). In order to find the optimal solution for (1), we need to define the conditions of optimality [15, 48–50]:

(4) ∂L∂w=0⟶w=∑i=1mαiyiϕxi∂L∂b=0⟶∑i=1mαiyi=0∂L∂ei=0⟶αi=Cei,∀i∂L∂αi=0⟶yiwTϕxi+b−1+ei=0,∀i.

Variables **w** and **e** can be eliminated and setting **y**, 1_*v*_, **e**, and **α** properly (see [15, 48–50] for details) we end up to a set of linear equations:

(5) $$\begin{array}{c} \left[\begin{array}{cc} 0 & y^{T}\\ y & \Omega +\frac{I}{C} \end{array}\right]\left[\begin{array}{c} b\\ \alpha \end{array}\right]=\left[\begin{array}{c} 0\\ 1_{v} \end{array}\right], \end{array}$$

where *Ω*_*ij*_ = *y*_*i*_ *y*_*j*_ *K*(**x**_*i*_, **x**_*j*_)  *i*, *j* = 1,2,…, *m*, and *K*(·, ·) is a kernel function [15, 48–50]. By solving **α** and *b* from (5), we obtain a classifier in the dual space:

(6) $$\begin{array}{c} y\left(\;x\;\right)=\mathrm{sign}\left[\;\overset{m}{\underset{i=1}{\sum }}\alpha_{i}y_{i}K\left(\;x,x_{i}\;\right)+b\;\right] \end{array}$$

which is equivalent to Vapnik's standard SVM formulation [48–50].

### 2.2. Multiclass Extensions of Least-Squares Support Vector Machines

#### 2.2.1. One-versus-All

One-versus-all (OVA) also known as one-versus-rest is the first and the most intuitive approach to extend SVM to concern multiclass problems (the number of classes in classification problem is higher than two). Rifkin and Klautau [43] and Galar et al. [41] made an extensive overview to OVA method and in [48] Least-Squares SVM was extended to multiclass cases. The basic idea behind OVA is very simple. If we have an *M* > 2 class problem, we train *M* binary SVM classifiers where each one of them separates one class from the rest. An advantage of this approach is the low number of classifiers but disadvantages are possible ties and are that all classifiers are required to be trained using all training data.

For instance, in our research, we have three classes (bad/good/semigood or 1/2/3, resp.) and, thus, we construct three classifiers “1-versus-rest,” “2-versus-rest,” and “3-versus-rest” and the classifier which gives the positive output assigns the predicted class label for test example. If “1-versus-rest” classifier, for example, gives an output of 1 and the rest of the classifiers give output “rest,” predicted class label will be 1 for test example. However, this approach consists of a problem when two or more classifiers give positive output or all classifiers give output “rest” for test example. Thus, we end up to a tie situation. In these cases, a common practice is to apply the so-called winner-takes-all principle where we need to compare the real outputs of OVA classifiers. In other words, we are looking for

(7) $$\begin{array}{c} arg\underset{i=1,2,\ldots ,M}{max}{\;f}_{i}\left(\;x\;\right), \end{array}$$

where *f*_*i*_ denotes the *i*th binary classifier which separates the class *i* from the rest and **x** is the test example. We used method given in [42] to solve possible ties. In [42], a 1-NN classifier was trained with the training data of tied classes and a predicted class label was solved using trained 1-NN classifier with Euclidean distance measure.

#### 2.2.2. One-versus-One

One-versus-one (OVO) or pairwise coupling [36, 37, 41, 42, 47] is another commonly used SVM extension. If we again have an *M* class classification problem, we construct a binary classifier for each class pair (*i*, *j*), where *i* ≠ *j* and *i* < *j* when class labels are converted to numbers. Thus, we have altogether *M*(*M* − 1)/2 classifiers. A problem now becomes how to combine the results from individual binary SVM classifiers. Galar et al. [41] listed different methods for aggregations in OVO such as weighted voting strategy and nesting one-versus-one method.

The most simplest way to obtain the predicted class label is to use majority voting [44] where we count the votes given by each classifier and assign predicted class label to be that class which has the most votes. However, like in OVA, voting is not always unambiguously determined and a tie might occur and some tie breaking rule must be applied. In this paper, we used, as in the case of OVA, a 1-NN classifier as a tie solver. The actual tie breaking procedure goes as follows:

(1) Collect the training data from classes that occurred in a tie.
(2) Train a 1-NN classifier using Euclidean metric and the training data obtained in step (1).
(3) Classify a test example that occurred in a tie using the trained 1-NN classifier in step (2).

#### 2.2.3. DAGSVM

Directed Acyclic Graph Support Vector Machines (DAGSVMs) use Decision Directed Acyclic Graph (DDAG) structure. DAGSVM was introduced by Platt et al. [16]. There is a great similarity between DAGSVM and OVO since the training phase is the same for both methods. DDAG consists of *M*(*M* − 1)/2 nodes and each one of nodes has an SVM classifier [16, 38]. However, testing differs compared to OVO since it begins at the root node and continues via directed edges until a leaf node (predicted class label) is reached [38]. Altogether, *M* − 1 comparisons are needed in the testing for an *M* class classification task. When in OVO ties can be a problem, in DAGSVM ties are not a problem since one-by-one classes are eliminated based on the decision of an SVM classifier.

**Figure 1:**
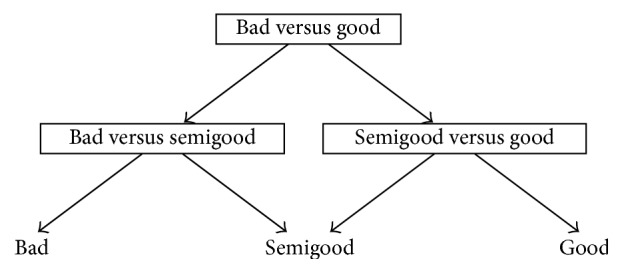
Structure 1 for automated quality identification of human iPSC colony images.

**Figure 2 fig2:**
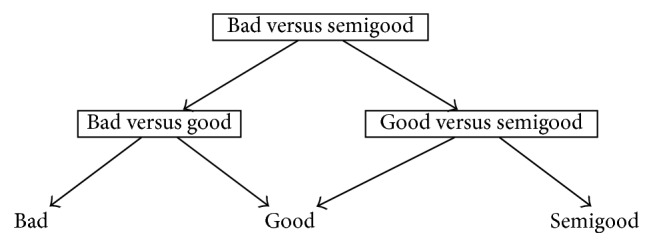
Structure 2 for automated quality identification of human iPSC colony images.

**Figure 3:**
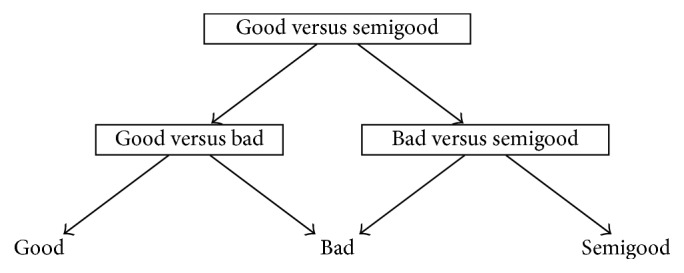
Structure 3 for automated quality identification of human iPSC colony images.

One reason behind the development of DAGSVM was to tackle the problem related to unclassifiable regions where ties occur [41]. Although the ties are not anymore a problem in DAGSVM, a new problem is encountered. DDAG structure can be constructed with various ways and each one of them may produce different classification results [14] so the important question is which order is the best one from the classification point of view. In a general case when the number of classes is high, it is impossible in practice to test all possible orders [14, 38]. However, since we have only three classes in our dataset, it is possible to construct all the different orders and to determine the best choice for our application. Figures 1 [Fig fig2]–3 show the DAGSVM structures what have been used in this study.

#### 2.2.4. Binary Tree Support Vector Machines

Lorena et al. [55] made an extensive review on how to combine binary classifiers in multiclass problems. One possible solution is to use tree structures in classification. Compared to general DAG structures, trees have a simpler architecture and their use in classification tasks has gained popularity among practitioners and researchers. A central question, when trees are used in classification, is how to construct a tree. Lei and Govindaraju [56] used half-against-half technique [57, 58] together with hierarchical clustering whereas Schwenker and Palm [46] applied confusion classes to define the binary partitions in a tree structure. Frank and Kramer [45, 55] stated that there are ∏_*i*=3_^*M*^2*i* − 3 possible ways to construct a tree for a multiclass problem.

**Figure 4:**
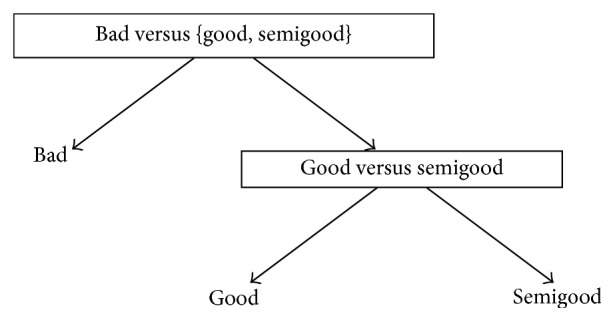
Structure 4 for automated quality identification of human iPSC colony images.

**Figure 5 fig5:**
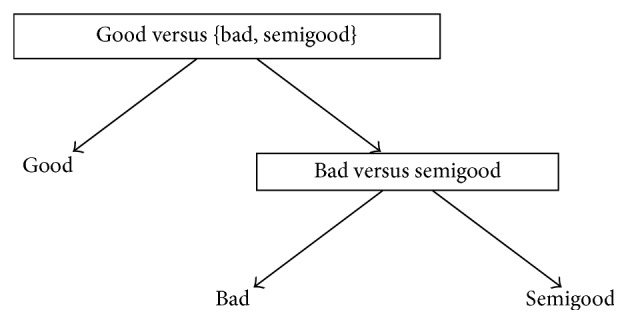
Structure 5 for automated quality identification of human iPSC colony images.

**Figure 6:**
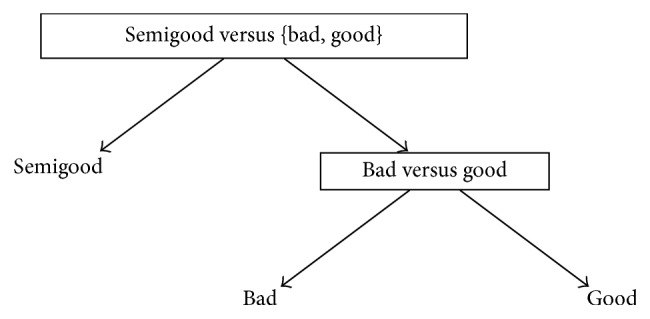
Structure 6 for automated quality identification of human iPSC colony images.

One possible binary tree structure is to reformulate one-versus-all method. This approach was applied in [45, 59] with good results. Classification begins at the root node similarly as in DAGSVM and continues via directed edges. Each node eliminates one class from the classification and classifiers in nodes are trained by one-versus-all principle. In the best case scenario, classification can end in the root node but the worst case scenario is that we need *M* − 1 comparisons. The problem encountered in this approach is again the question in which order the classes should be eliminated in order to achieve the best possible classification performance. Fortunately, due to the low number of classes in our application, we were able to test all possible orders and the corresponding trees can bee seen from Figures 4 [Fig fig5]–6. However, in a general *M* class case where *M* is very large, it is impossible to go through all possible orders computationally and some intelligent selection method must be applied.

## 3. Dataset

### 3.1. Image Data Acquisition

Human induced pluripotent stem cells were used in this study and the colonies were photographed between days 5 and 7 of their weekly growth cycle [14, 15]. The reason behind the choice of days 5–7 is the requirement to gain better visualization of the iPSC colonies [14, 15]. The growing iPSC colonies were observed before taking the colony images. In observation of iPSCs, Nikon Eclipse TS100 inverted routine microscope with an attached heating plate was used [14, 15]. After visual observation, photographed iPSC colonies were categorized to one of the classes (good/semigood/bad) [14, 15]. In the imaging process, lighting and sharpness of an image were user-defined which might bring some variability between images [14, 15]. Nevertheless, the same expert took all the images which minimized the variability [14, 15]. Furthermore, settings were fixed during photographing sessions [14, 15].

Overall, images were taken during several sessions and it caused some minor differences in the images [14, 15]. Growing iPSC colonies were usually located in the center of the image, thus, giving the best visual condition [14, 15]. However, sometimes observed colony was located near the edge of the well which caused some distortion in the lightning [14, 15]. Image acquisition was performed using Imperx IGV-B1620M-KC000 camera which was mounted to the microscope and connected to a notebook equipped with JAI Camera Control Tool software [14, 15]. All the images were taken with 1608 × 1208 (width × height) resolution.

### 3.2. Dataset Description

**Figure 7:**
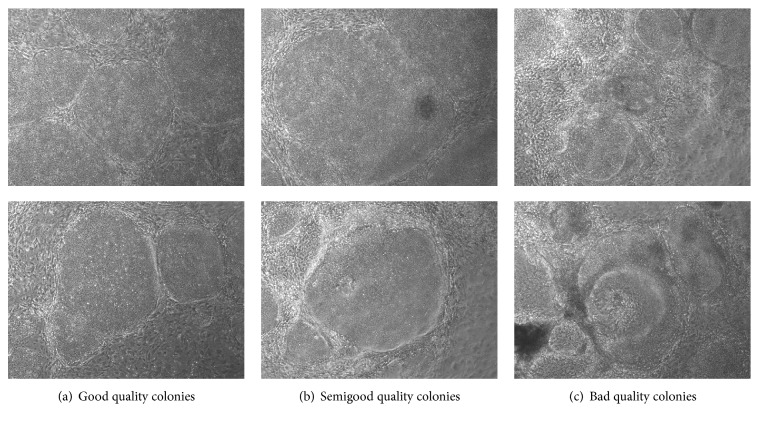
Example images of good, semigood, and bad quality iPSC colonies. The images have been scaled to have width and height of 1.5 in.

The study was approved by the ethical committee of Pirkanmaa Hospital District (R08070). iPSC lines were established with retroviruses encoding for OCT4, SOX2, KLF4, and MYC as described earlier [4]. All the cell lines had been characterized for their karyotypes and pluripotency as described earlier [60]. The colonies were categorized as good quality if they had rounded shape, translucent even color, and defined edges. Semigood quality colonies presented changes in color and structure, but still with clear edges, while bad quality colonies had partially lost the edge structure, vacuole could occasionally be observed and areas of three-dimensional structures were observed (see Figure 7).

An image dataset containing 173 images altogether was analyzed including colonies with good, semigood, and bad quality.  Table 1 indicates the accurate information of frequencies and proportions of the classes in dataset. All image data are anonymous and cannot be attached to any specific patient. All the images have been taken by the same expert who also determined the true class label for each image as was explained in Section 3.1. Figure 7 shows two example images from each class.

## 4. Design of Experiments

### 4.1. Feature Extraction

Feature extraction from images has attracted researchers for a long time and there is a huge amount of research related to this topic. For instance, histograms are used in image classification (see [21]) and Local Binary Patterns [20, 61, 62] is a commonly used method. We used another well-known feature extraction method called Scaled Invariant Feature Transformation (SIFT) which was developed by Lowe [17, 18, 20]. Lowe [17] presents four steps with the corresponding descriptions for computing the SIFT features and these steps are as follows:

1. The first step is scale-space extrema detection where possible interest points, which are invariant to scale and orientation, are detected [17]. Search is implemented using a difference-of-Gaussian function [17].
2. The second step is keypoint localization where a detailed model is fitted to the nearby data for location, scale, and ratio of principal curvatures [17]. With the help of this information, unstable keypoint candidates can be excluded [17].
3. The third step is orientation assignment. In this step, for each keypoint local image gradients are evaluated and based on this information all possible orientations are assigned for keypoints [17]. By this means, keypoints obtain rotational invariant property [17].
4. The last step is to compute keypoint descriptors. In the previous steps location, scale and orientation of keypoints have been found. Determination of descriptors is made by measuring the local image gradients at the fixed scale in the environment of each keypoint [17]. Finally, local gradients are transformed so that they are highly distinctive and still invariant as possible to any other possible change [17].

Overall, according to [17], “SIFT transforms image data into scale-invariant coordinates relative to local features.” More details concerning all the aforementioned stages can be found in [17]. We used the Matlab implementation of VLFeat 0.9.18 [63] when extracting the SIFT frames and their corresponding descriptors from the images. We used the default values in extraction of SIFT features. Each one of the descriptors is described as a 128-dimensional vector and the dimension comes from Lowe's algorithm. After extracting SIFT descriptors from the images, a question arises what to do with them and how we can use them in classification. It is not unusual that, from an image, for instance, ten thousand descriptors are obtained and when we have a large image collection, the number of descriptors in total becomes very high. A common strategy is to apply bag-of-features (BoF) [64–66] approach which originates from bag-of-words strategy used in natural language processing and information retrieval. Csurka et al. [65] define four stages for image classification when BoF is applied to image classification. These stages are [64, 65]

1. feature extraction and representation,
2. codebook construction,
3. BoF representation of images,
4. training of learning algorithm.

From the stages, codebook construction is computationally tedious because in this phase the descriptors from the training set images are collected together and they are clustered using *K*-means algorithm or other clustering algorithms. Centroids of the clusters are codewords and with the use of centroids we can solve how many descriptors from each image belong to each of the clusters and, thus, we can obtain a histogram for every image what can be used in training of a learning algorithm. The next step is that from the images of a test set SIFT descriptors are extracted and they are clustered by means of centroids. Thus, a histogram presentation can be produced for the test set of images and they can be used in classification.

However, BoF approach has a significant weakness. Since the number of clusters define the number of features, we need to find the optimal value of *K*. This again leads to a situation where we need to repeat the *K*-means algorithm several times and when the number of descriptors is hundreds of thousands or even millions we require a lot of computational power.

We present a simple way to bypass the clustering phase. Our procedure goes as follows:

1. Repeat steps (2)–(4) for all images.
2. Extract SIFT descriptors from an image.
3. Compute a mean SIFT descriptor from descriptors gained in step (2). By this means, one obtains one 128-dimensional vector.
4. Use the average descriptor obtained in step (3) in classification as a feature vector.

In other words, we evaluate a mean SIFT descriptor for each image and the obtained mean descriptor can be used in classification.

### 4.2. Classification Procedure

#### 4.2.1. Performance Measures

We selected three performance measures for this paper. Firstly, we used true positive (TP) measure which indicates the number of correctly classified samples from specific class. Secondly, we used true positive rate (TPR) in percentage which tells the proportion how well the specific class was classified (0% is the minimum and 100% is the maximum). Thirdly, we applied accuracy (%) which is defined as a trace of confusion matrix divided by the sum of all elements in a confusion matrix and it shows the overall performance [14, 15].

#### 4.2.2. *k*-Nearest Neighbor

*k*-nearest neighbor method (hereafter referred to as *k*-NN) [22, 24, 25, 67] is a commonly used baseline classification method. There are three main parameters to be considered when *k*-NN is used in classification and these are

(1) the value of *k*,
(2) distance measure,
(3) distance weighting function.

All of these parameters are user-defined and there is no universal rule how to define optimal parameter combination since they are data dependent. For this research, we selected *k* ∈ {1,3,…, 23} when the total number of tested *k* values was 12. The odd *k* values were selected in order to decrease the probability of ties. If a tie in a *k*-NN classifier occurred, it was solved by a random choice between tied classes. A huge variety of distance measures are available in the literature and we tested seven of them. The chosen measures were as follows:

1. Chebyshev.
2. Cityblock (also known as Manhattan measure).
3. Correlation.
4. Cosine.
5. Euclidean.
6. Standardized Euclidean.
7. Spearman.

And these are the same as used in [14]. To the last parameter, distance weighting function, we also applied the same alternatives as in [14] and these were as follows:

1. Equal weighting.
2. Inverse weighting.
3. Squared inverse weighting.

From the parameters, distance and weighting function can be fixed but the optimal *k* value must be estimated. Common method for *k* value estimation is to use cross-validation technique [23, 25] and the extreme variation of it is leave-one-out (LOO) method known to be suitable for rather small datasets as ours. In this paper, we performed *k*-NN classification using nested cross-validation [68–71] and, more specifically, nested leave-one-out (NLOO) method which consists of a two inner loops where the outer loop is for model assessment and the inner loop is for model selection.

NLOO is the most time-consuming variant for parameter value estimation but the advantage lies especially in the maximization of training set size which is valuable when we are dealing with relatively small datasets. Basically, with the help of NLOO, we can estimate optimal parameter values for all examples in a dataset separately. If we had a dataset with thousands or tens of thousands examples, we could simply use the hold-out method and apply 10-fold cross-validation to a training set, for example, in order to find optimal parameter values. NLOO would be very inefficient to use in the case of “big data.” NLOO procedure used in this paper can be described with the following steps and notice that NLOO has the consequence that there might not be one specific optimal *k* value for all examples but the *k* value might vary:

(1) Let *N* = 173, *i* ∈ {1,2,…, 173}, and *D* is the dataset.
(2) Repeat the following steps for all *i* ∈ {1,2,…, 173}. Let Test_*i*_ be the *i*th example excluded from *D*. Hence, the rest of *N* − 1 samples form the training set Train_*i*_.
(3) Perform LOO procedure for Train_*i*_ with the following way:
  (a) Repeat steps (b)–(d) in each LOO round.
  (b) Do a *z*-score standardization for the smaller training set and scale the validation example using the values of ***μ*** and **σ** obtained from the smaller training set.
  (c) Train *k*-NN classifier using specific *k* value and standardized training data obtained in step (b).
  (d) Predict the class label for the validation example using the trained *k*-NN classifier.
  (e) When the last LOO round has been performed, evaluate accuracy of Train_*i*_ for the tested *k* value.
(4) When LOO procedure has been repeated with all *k* values in Train_*i*_, select the *k* value which obtained the highest accuracy.
(5) When in step (4) the optimal *k* value for Train_*i*_ has been found, do *z*-score standardization for Train_*i*_ and scale Test_*i*_ using ***μ*** and **σ** obtained from Train_*i*_.
(6) Train the *k*-NN classifier using Train_*i*_ and predict class label for Test_*i*_.
(7) When all Test_*i*_, *i* = 1,2,…, 173, have been predicted, evaluate performance measures specified in Section 4.2.1.

#### 4.2.3. Multiclass Support Vector Machines

In Section 2.2, multiclass extensions of SVM were described and now we present the common classification procedure what was used in this paper. Support Vector Machines include parameters to be estimated like *k*-NN. However, the number of parameters depends on the kernel. We tested altogether seven kernels: the linear, polynomial kernels having degrees from 2 to 6, and the Gaussian Radial Basis Function (RBF) kernel. A common parameter for all kernels is the regularization parameter *C* (also known as “boxconstraint”) and for RBF there is also another parameter, *σ*, to be estimated which is the width of Gaussian function.

We decided that parameter value spaces were the same for *C* and *σ*; that is, *C*, *σ* ∈ {2^−15^, 2^−14^,…, 2^15^}. Thus, 31 parameter values were tested for kernels other than RBF and 961 combinations of (*C*, *σ*) were tested for the RBF kernel. We used Least-Squares Support Vector Machines [48–50] in our implementations of multiclass SVMs and the possible ties in OVA and OVO were solved by using the 1-NN classifier with Euclidean measure as was done in [42]. We used the autoscale property in training of a binary SVM classifier which means that the training data of an SVM classifier was *z*-score standardized and in the testing phase every SVM classifier scales test/validation example based on ***μ*** and **σ** obtained from training data. The autoscaling property was used in order to have the consistent classification procedure for all classification methods. Furthermore, all SVM classifiers in a multiclass extension were trained with the same parameter values as in [37].

The actual classification and parameter value search was performed by using NLOO approach described as follows:

(1) Let *N* = 173, *i* ∈ {1,2,…, 173}, and *D* is the dataset.
(2) Repeat the following steps for all *i* ∈ {1,2,…, 173}. Let Test_*i*_ be the *i*th example excluded from *D*. Hence, the rest of *N* − 1 samples form the training set Train_*i*_.
(3) Perform LOO procedure for Train_*i*_ with the following way:
  (a) Repeat steps (b)-(c) in each LOO round of Train_*i*_.
  (b) Train individual SVM classifiers using the smaller training data, specified kernel function, and parameter value *C* or combination (*C*, *σ*) in the case of RBF kernel.
  (c) Predict the class label for the validation example using trained SVM classifiers.
  (d) When the last LOO round has been performed, evaluate accuracy of Train_*i*_ for the tested parameter value (combination).
(4) When LOO procedure has been repeated with all values of *C* or combinations (*C*, *σ*) in Train_*i*_, select the parameter value (combination) which obtained the highest accuracy.
(5) When in step (4) the optimal *C* or (*C*, *σ*) for Train_*i*_ has been found, train SVM classifiers again using full Train_*i*_ and predict the class label for Test_*i*_.
(6) When all Test_*i*_, *i* = 1,2,…, 173, have been predicted, evaluate performance measures specified in Section 4.2.1.

The consequence of NLOO method is that we cannot find one specific parameter value or parameter value combination but the optimal values may vary in the case of each test example.

#### 4.2.4. Other Classification Methods

Since the other classification methods (classification tree, linear discriminant analysis, naïve Bayes and its variants, and multinomial logistic regression) used in this research did not require any parameter value estimation, we were able to use simpler LOO approach in classification. We also tested quadratic discriminant analysis (QDA) [23] and discriminant analysis using Mahalanobis distance [35] but both of these methods could not be evaluated since some of the covariance matrices in a training set were not positively defined and positively definiteness is a requirement for calculating the inverse of a covariance matrix which is needed for QDA and Mahalanobis distance calculations. The classification procedure can be explained in detail with the following way:

(1) Let *N* = 173, *i* ∈ {1,2,…, 173}, and *D* is the dataset.
(2) Repeat the following steps for all *i* ∈ {1,2,…, 173}. Let Test_*i*_ be the *i*th example excluded from *D*. Hence, the rest of *N* − 1 samples form the training set Train_*i*_.
(3) Do a *z*-score standardization for Train_*i*_ and scale Test_*i*_ based on ***μ*** and **σ** obtained from Train_*i*_.
(4) Train a classifier using Train_*i*_ and predict the class label of Test_*i*_ using trained classifier.
(5) When all Test_*i*_, *i* = 1,2,…, 173, have been predicted, evaluate performance measures specified in Section 4.2.1.

We made all the tests and implementations of multiclass extensions of LS-SVM with Matlab 2014a together with Image Processing Toolbox, Parallel Computing Toolbox, and Statistics Toolbox. In SIFT feature extraction, we used VLFeat 0.9.18 [63].

## 5. Results

In the tables, we have emphasized the best result or results in a tie situation in order to facilitate the analysis for a reader. When analyzing the results, it is good to keep in mind that in our preliminary researches [14, 15] with smaller dataset the best accuracy was 55%.

OVA approach has been suggested to be as good as other multiclass methods when classifiers such as SVMs are properly tuned [43]. When looking into the OVA results in Table 2, we notice immediately two facts. Firstly, the RBF kernel obtained the highest accuracy (60.1%) and, secondly, semigood class was the most difficult class to be recognized since the TPRs were always lower compared to corresponding TPRs of classes good and bad. One possible reason behind the difficulty of classification of class semigood might be that semigood class can be considered as a transition phase and it might consist of very heterogeneous colonies. Some of the colonies may include only small changes and be very close to good colonies whereas some other colonies might be closer to class bad colonies depending on the situation. An answer to the other questions related to the performance of the RBF kernel might lie in the OVA strategy itself. When SVM classifiers are trained in OVA, we need to use all training data in every binary classifier which might complicate the separation of classes and, thus, the simpler kernels do not perform well. Overall, the linear, quadratic, and RBF kernels were the best choices when using OVA since they were the only ones which achieved above 50% accuracy. The best TPs and TPRs for each class were 25 (61.0%) (class bad with the RBF and 5th degree polynomial kernels), 51 (68.9%) (class good with the linear kernel), and 29 (50.0%) (class semigood with the RBF kernel).

The results of OVO in Table 2 differ from OVA results. Firstly, now in each class, the linear kernel was the best one obtaining the highest TPs and TPRs and gained the highest accuracy (60.7%). The TPs and TPRs were 28 (68.3%) for class bad, 52 (70.3%) for class good, and 25 (43.1%) for class semigood. Other kernels with above 50.0% accuracies were the 3rd degree polynomial and RBF kernels. Now, the results had slightly different trend compared to OVA results and it might be explained with the fact that in OVO SVM classifiers are trained only with training data of *i*th and *j*th classes whereas in OVA results all SVM classifiers are trained using a full training set.

Table 3 shows the results of DAGSVM with different structures and kernel functions. If we go through the results of different orders one by one, we notice similarities with the OVO results. This is, of course, natural since DAGSVM has the same training phase as OVO but the difference appears in testing. In structure 1 (see Figure 1), in the root node, classes bad and good were separated. From the accuracies in structure 1, the linear (60.7%), 3rd degree polynomial (50.3%), and RBF (59.5%) kernel were the best ones as also in OVO results. TPRs above 70.0% were achieved only by the linear kernel in the case of classes bad and good. Class semigood was usually the worst (the highest TPR was 46.6% with 3rd degree polynomial kernel) recognized class, but when the degree of polynomial kernel function increased, TPRs in class semigood stayed relatively stable whereas in other classes TPRs dropped significantly.

In structure 2 (see Figure 2), where the root node separated classes bad and semigood from each other, the best results were improved compared to structure 1 results. Now, the best accuracies were gained by the same kernels as in OVO and structure 1 case. However, from structure 1, the accuracies of the linear (61.8%), 3rd degree polynomial (51.4%), and RBF (61.3%) kernels were improved above 1%. Respectively, TPs and TPRs for the linear kernel were 29 (70.7%) (class bad), 54 (73.0%) (class good), and 24 (41.4%) (class semigood). For the RBF, the only difference compared to the linear kernel TPs or TPRs was in the case of class good where TP and TPR were 53 (71.6%). Moreover, in class semigood, TPRs were not so stable as in structure 1 within different kernels.

The last DAGSVM structure (see Figure 3) obtained the lowest maximum accuracy within all DAGSVM structures. In this structure, classes good and semigood were separated in root node. Nevertheless, the same kernels obtained the best results as in previous structures. The linear kernel achieved 59.0% accuracy and the RBF 54.9% accuracy, respectively. The quadratic and 3rd degree polynomial kernels yielded 48.6% accuracy. From the TPRs, the only case where 70.0% was exceeded was for class good with the linear kernel 52 (70.3%). Otherwise, all TPRs were left below 67.0%. For classes bad and semigood, TPs and TPRs of the linear kernel were 27 (65.9%) and 23 (39.7%). Similarly, as in structure 2, semigood TPRs had a great dispersion between kernels. One reason behind this might be in the difference of class sizes of good and semigood. In structure 2 where the best accuracy was achieved, the balance between the class sizes in root node was also good.

Table 4 presents the results of binary tree SVMs of Figures 4 [Fig fig5]–6. All structures represent OVA method described in a binary tree formulation. Structure 4 (see Figure 4) separated class bad from the rest of the classes in root node. Now, the highest accuracy (57.2%) was achieved by the linear kernel again and the TPs and TPRs were 28 (68.3%) (class bad), 52 (70.3%) (class good), and 19 (32.8%) (class semigood). It must be noticed that the TP and TPR of the linear kernel in class bad were the highest among structures 4–6. Other kernels which reached the 50.0% limit in accuracy were the quadratic and RBF kernels. The overall results of structure 4 are comparable with OVA results and reflect the difficulty of separating one class from the rest in this application. With many kernels, class good was recognized with the best TPR and this is somehow a natural situation since class good was the biggest class in a dataset. One obvious reason might also be that class good is just better separable class in the feature space than other classes.

For structure 5 (see Figure 5) where class good was separated from other classes in the root node, the results did not change a lot from structure 4 results. However, there are some interesting differences. Firstly, the highest accuracy (60.1%) was gained using this structure and with the linear kernel. A noticeable detail is that this accuracy was the same as the topmost accuracy in OVA results. Secondly, the best TP (59) and TPR (79.7%) of class good were yielded by the linear kernel within all structures 4–6. For the class semigood, which usually was the most difficult class to be classified, the same trend continued also when structure 5 was used. Thirdly, the highest TP(R) combination 26 (44.8%) was achieved by the 3rd degree polynomial kernel and the corresponding accuracy was 53.2%. These are clear differences from structure 4 results. The RBF kernel was a runner-up as in many cases before.

For the last binary tree structure, structure 6 (see Figure 6), the general level of results was lower compared to structures 4 and 5 results. All the accuracies were left below 50.0% and the best accuracy (48.6%) was obtained by the RBF kernel and not by the linear kernel as usual. If we fix a kernel and look for the TPRs from all classes, we notice that the level of TPRs is more balanced in a bad way compared to many other cases before. This, however, affects directly the results decreasingly. Class good, for instance, was recognized below 60.0% TPRs with all kernels whereas with other structures over 70.0% and nearly 80.0% TPRs were achieved. Moreover, in class bad, all TPRs were left below 50.0% as well as in class semigood. Again, one reason behind the poor performance of structure 6 may be that in the root node we were separating class semigood from the other classes. Class semigood seems to be confusing class in our dataset and it is easily mixed up with the other classes. More details can be found from Table 4.

The move from multiclass SVMs into other classification methods did not bring any global improvement to the results. From the accuracies, naïve Bayes (NB) with and without kernel smoothing density achieved accuracy of 52.6% which was the best one in Table 5. This, however, is not a satisfactory result. When considering the classwise TPs and TPRs, classification tree was the best alternative for classes bad and semigood having TP(R)s 20 (48.8%) and 19 (32.8%). For class good topmost TP(R) were obtained by the traditional NB and the results were 61 (82.4%). This was the first time when a TPR value reached above 80.0% limit. An explanation for NB result in class good may be that NB is a classifier which uses probabilities in classification phase and class good is the largest class in a dataset so it has also the highest a priori probability.

Table 6 presents the results of *k*-NN classifiers with different distance measures and weighting combinations. Accuracy column shows that there are three accuracies above 60.0% and these were gained by the Euclidean measure and equal weighting (62.4%), Euclidean measure with inverse weighting (60.7%), and standardized Euclidean measure with inverse weighting (60.7%). Accuracy of 62.4% is the best one throughout all the classification methods used in this paper and it improved the accuracy of our previous researches [14, 15] around 8.0%. However, the accuracy of 55.0% was yielded using smaller dataset compared to dataset which is used in this paper, so the improvement is even more valuable. Overall, *k*-NN succeeded well in the classification. If we exclude two distance measure and weighting combinations, all other accuracies were above 50.0% whereas several SVM results did not achieve above 50.0% accuracy. A closer look to the TPs and TPRs reveals that Euclidean measure was also a good choice for classes bad and semigood since the topmost results 25 (61.0%) and 29 (50.0%) were achieved using it. For class good, cosine measure together with inverse weighting obtained the highest TP and TPR being 62 (83.8%). These were the best ones also in the whole paper. Table 6 also shows that class good was generally the best classified class in the dataset and class semigood was usually the most difficult class to be recognized.

## 6. Discussion and Conclusions

This study focused on automated identification of the quality of iPSC colony images. The classification task was a multiclass problem with three possible classes (good/semigood/bad) for the iPSC colony images. The motivation behind the paper is both practical and scientific. iPS cell technology will be in the near future a standard method in drug and toxicology screens in vitro and for creating disease models in culture [3]. In long-term perspective, iPS cell technology will probably be used also for tissue repairing and the possibilities of iPSC technology are enormous [3]. From the practical point of view, iPSCs cannot be exploited for future needs without the help of image analysis and classification techniques.

When iPSCs are differentiating, the growing process of colonies must have automated monitoring because of at least three reasons. Firstly, we need to ensure that the newly reprogrammed iPSC colonies have proper quality and structure. Secondly, the quality of the iPSC colonies after multiple passages must remain good without signs of spontaneous differentiation. Thirdly, when the number of growing iPSC colonies is large which means thousands or millions of colonies, it is impossible to manage the quality control manually and, hence, automation of this process is inevitable.

The aim of this paper was to give new perspective to this difficult identification problem by using SIFT descriptors in feature extraction and to present a simple way to handle these descriptors. Moreover, we wanted to give an extensive overview to different classification methods. A special focus was given on DAGSVMs and binary tree LS-SVMs where the crucial question was to find the right order to construct the graph or tree since different orders might always give different results. As a result, different constructions gave different results and the differences were clear. Overall, we performed over 80 test arrangements and made thorough parameter value searches for SVMs and *k*-NN classifiers.

The best result was obtained by *k*-NN classifier with Euclidean measure and equal weighting having the accuracy of 62.4%. The accuracy itself might feel low but we have gained significant improvement from the earlier researches [14, 15] where a smaller image collection was examined and intensity histograms were used as a feature. Our earlier publications already showed how difficult problem this is and the differences between images and classes are very small.

Just by watching the best accuracy, it is obvious that more research is needed. At the current stage, classifier which performs with a bit over 60% accuracy can work as a decision supporting tool for personnel. However, in order to move large-scale better accuracy must be obtained. When taking into account that this paper is a preliminary study with the extended dataset, we have gained promising results and are able to take next steps further in our research. Since with many classification methods class good was recognized well, it gives an idea that classes semigood and bad could be merged and the classification task would reduce to binary classification problem. This could be a good idea because in the end our aim from the practical point of view is to separate good colonies from the rest since good hiPSC colonies can only be used in applications.

Although we have obtained improvement to our results, we have still many open questions. From the classification perspective, artificial neural networks have not been used for this problem and that is why a thorough examination of different ANN learning algorithms is needed as in the previous work with benthic macroinvertebrate image classification [72]. An important question related to the simple descriptor handling method is the use of feature selection methods. An obvious continuation is to apply, for instance, Scatter method [73] to this averaged SIFT descriptor data and to examine how it affects classification results. Moreover, we need to examine other sophisticated feature selection methods.

An essential question related to feature extraction is to use other texture descriptors such as Local Binary Patterns, Local Intensity Order Patterns, dense SIFT, intensity histograms, and Histogram of Oriented Gradients, for iPSC colony classification problem. Moreover, BoF approach must be tested in the future with aforementioned textural descriptors and with normal SIFT descriptors. A good example on comparison of different texture descriptors can be found from [74, 75]. Although automated quality identification of human iPSC colony images has shown to be a real challenge for the computational methods, we have gained improvement and we are fully convinced that the technical challenges will be overcome in future research.

## Figures and Tables

**Table 1:** Frequencies and percentages of classes in dataset.

Class	Frequencies	Percentages
Good	74	42.8%
Semigood	58	33.5%
Bad	41	23.7%

Total	173	100.0%

**Table 2:** Results of one-versus-one and one-versus-all methods when different kernels were used. True positive rates (%) are given in parentheses and accuracy (%) can be found from the last column.

Kernel/class	Bad	Good	Semigood	ACC
One-versus-all				
Linear	22 (53.7%)	**51 (68.9%) **	18 (31.0%)	52.6%
Polynomial *deg* = 2	23 (56.1%)	50 (67.6%)	26 (44.8%)	57.2%
Polynomial *deg* = 3	18 (43.9%)	43 (58.1%)	22 (37.9%)	48.0%
Polynomial *deg* = 4	23 (56.1%)	39 (52.7%)	20 (34.5%)	47.4%
Polynomial *deg* = 5	**25 (61.0%) **	34 (45.9%)	17 (29.3%)	43.9%
Polynomial *deg* = 6	23 (56.1%)	33 (44.6%)	18 (31.0%)	42.8%
RBF	**25 (61.0%) **	50 (67.6%)	**29 (50.0%) **	**60.1% **

One-versus-one				
Linear	**28 (68.3%) **	**52 (70.3%) **	**25 (43.1%) **	**60.7% **
Polynomial *deg* = 2	21 (51.2%)	46 (62.2%)	15 (25.9%)	47.4%
Polynomial *deg* = 3	20 (48.8%)	45 (60.8%)	24 (41.4%)	51.4%
Polynomial *deg* = 4	14 (34.1%)	41 (55.4%)	21 (36.2%)	43.9%
Polynomial *deg* = 5	24 (58.5%)	32 (43.2%)	21 (36.2%)	44.5%
Polynomial *deg* = 6	19 (46.3%)	39 (52.7%)	17 (29.3%)	43.4%
RBF	27 (65.9%)	50 (67.6%)	24 (41.4%)	58.4%

**Table 3:** Results of structures 1–3 given in Figures 1 [Fig fig2]–3 when different kernels were used. True positive rates (%) are given in parentheses and accuracy (%) can be found from the last column.

Kernel/class	Bad	Good	Semigood	ACC
Structure 1				
Linear	**29 (70.7%) **	52 (70.3%)	24 (41.4%)	60.7%
Polynomial *deg* = 2	21 (51.2%)	42 (56.8%)	19 (32.8%)	47.4%
Polynomial *deg* = 3	20 (48.8%)	40 (54.1%)	**27 (46.6%) **	50.3%
Polynomial *deg* = 4	14 (34.1%)	38 (51.4%)	26 (44.8%)	45.1%
Polynomial *deg* = 5	19 (46.3%)	26 (35.1%)	26 (44.8%)	41.0%
Polynomial *deg* = 6	15 (36.6%)	26 (35.1%)	24 (41.4%)	37.6%
RBF	28 (68.3%)	51 (68.9%)	24 (41.4%)	59.5%

Structure 2				
Linear	**29 (70.7%) **	**54 (73.0%) **	24 (41.4%)	**61.8% **
Polynomial *deg* = 2	20 (48.8%)	43 (58.1%)	16 (27.6%)	45.7%
Polynomial *deg* = 3	20 (48.8%)	46 (62.2%)	23 (39.7%)	51.4%
Polynomial *deg* = 4	13 (31.7%)	44 (59.5%)	15 (25.9%)	41.6%
Polynomial *deg* = 5	19 (46.3%)	37 (50.0%)	18 (31.0%)	42.8%
Polynomial *deg* = 6	15 (36.6%)	43 (58.1%)	12 (20.7%)	40.5%
RBF	**29 (70.7%) **	53 (71.6%)	24 (41.4%)	61.3%

Structure 3				
Linear	27 (65.9%)	52 (70.3%)	23 (39.7%)	59.0%
Polynomial *deg* = 2	22 (53.7%)	46 (62.2%)	16 (27.6%)	48.6%
Polynomial *deg* = 3	20 (48.8%)	40 (54.1%)	24 (41.4%)	48.6%
Polynomial *deg* = 4	19 (46.3%)	38 (51.4%)	17 (29.3%)	42.8%
Polynomial *deg* = 5	26 (63.4%)	26 (35.1%)	18 (31.0%)	40.5%
Polynomial *deg* = 6	22 (53.7%)	26 (35.1%)	12 (20.7%)	34.7%
RBF	25 (61.0%)	49 (66.2%)	21 (36.2%)	54.9%

**Table 4:** Results of structures 4–6 given in Figures 4 [Fig fig5]–6 when different kernels were used. True positive rates (%) are given in parentheses and accuracy (%) can be found from the last column.

Kernel/class	Bad	Good	Semigood	ACC
		Structure 4		
Linear	**28 (68.3%) **	52 (70.3%)	19 (32.8%)	57.2%
Polynomial *deg* = 2	25 (61.0%)	45 (60.8%)	19 (32.8%)	51.4%
Polynomial *deg* = 3	18 (43.9%)	42 (56.8%)	19 (32.8%)	45.7%
Polynomial *deg* = 4	23 (56.1%)	35 (47.3%)	19 (32.8%)	44.5%
Polynomial *deg* = 5	23 (56.1%)	28 (37.8%)	14 (24.1%)	37.6%
Polynomial *deg* = 6	23 (56.1%)	24 (32.4%)	15 (25.9%)	35.8%
RBF	22 (53.7%)	48 (64.9%)	23 (39.7%)	53.8%

Structure 5				
Linear	24 (58.5%)	**59 (79.7%) **	21 (36.2%)	**60.1% **
Polynomial *deg* = 2	18 (43.9%)	44 (59.5%)	24 (41.4%)	49.7%
Polynomial *deg* = 3	20 (48.8%)	46 (62.2%)	**26 (44.8%) **	53.2%
Polynomial *deg* = 4	16 (39.0%)	41 (55.4%)	23 (39.7%)	46.2%
Polynomial *deg* = 5	19 (46.3%)	37 (50.0%)	21 (36.2%)	44.5%
Polynomial *deg* = 6	15 (36.6%)	38 (51.4%)	20 (34.5%)	42.2%
RBF	20 (48.8%)	52 (70.3%)	21 (36.2%)	53.8%

Structure 6				
Linear	20 (48.8%)	38 (51.4%)	24 (41.4%)	47.4%
Polynomial *deg* = 2	15 (36.6%)	38 (51.4%)	**26 (44.8%) **	45.7%
Polynomial *deg* = 3	18 (43.9%)	34 (45.9%)	20 (34.5%)	41.6%
Polynomial *deg* = 4	17 (41.5%)	37 (50.0%)	20 (34.5%)	42.8%
Polynomial *deg* = 5	21 (51.2%)	28 (37.8%)	20 (34.5%)	39.9%
Polynomial *deg* = 6	18 (43.9%)	22 (29.7%)	23 (39.7%)	36.4%
RBF	19 (46.3%)	43 (58.1%)	22 (37.9%)	48.6%

**Table 5:** Results of classification tree, linear discriminant analysis, multinomial logistic regression, and naïve Bayes variants. True positive rates (%) are given in parentheses and accuracy (%) can be found from the last column.

Method/class	Bad	Good	Semigood	ACC
Classification tree	**20 (48.8%)**	50 (67.6%)	**19 (32.8%)**	51.4%
Linear discriminant analysis	19 (46.3%)	35 (47.3%)	16 (27.6%)	40.5%
Multinomial logistic regression	17 (41.5%)	32 (43.2%)	**19 (32.8%)**	39.3%
Naïve Bayes	16 (39.0%)	**61 (82.4%)**	14 (24.1%)	**52.6%**
Naïve Bayes with kernel smoothing density estimation and normal kernel	18 (43.9%)	59 (79.7%)	14 (24.1%)	**52.6%**
Naïve Bayes with kernel smoothing density estimation and box kernel	12 (29.3%)	56 (75.7%)	11 (19.0%)	45.7%
Naïve Bayes with kernel smoothing density estimation and Epanechnikov kernel	13 (31.7%)	57 (77.0%)	11 (19.0%)	46.8%
Naïve Bayes with kernel smoothing density estimation and triangle kernel	13 (31.7%)	56 (75.7%)	12 (20.7%)	46.8%

**Table 6:** Results of *k*-NN with different weighting and measure combinations. True positive rates (%) are given in parentheses and accuracy (%) can be found from the last column.

Measure and weighting combination/class	Bad	Good	Semigood	ACC
Chebyshev/equal weights	18 (43.9%)	52 (70.3%)	26 (44.8%)	55.5%
Chebyshev/inverse weights	19 (46.3%)	53 (71.6%)	27 (46.6%)	57.2%
Chebyshev/squared inverse weights	17 (41.5%)	53 (71.6%)	27 (46.6%)	56.1%
Cityblock/equal weights	22 (53.7%)	55 (74.3%)	24 (41.4%)	58.4%
Cityblock/inverse weights	23 (56.1%)	52 (70.3%)	27 (46.6%)	59.0%
Cityblock/squared inverse weights	20 (48.8%)	51 (68.9%)	22 (37.9%)	53.8%
Correlation/equal weights	17 (41.5%)	59 (79.7%)	16 (27.6%)	53.2%
Correlation/inverse weights	16 (39.0%)	54 (73.0%)	14 (24.1%)	48.6%
Correlation/squared inverse weights	22 (53.7%)	53 (71.6%)	17 (29.3%)	53.2%
Cosine/equal weights	19 (46.3%)	57 (77.0%)	14 (24.1%)	52.0%
Cosine/inverse weights	19 (46.3%)	**62 (83.8%)**	15 (25.9%)	55.5%
Cosine/squared inverse weights	21 (51.2%)	50 (67.6%)	14 (24.1%)	49.1%
Euclidean/equal weights	24 (58.5%)	55 (74.3%)	**29 (50.0%)**	**62.4%**
Euclidean/inverse weights	**25 (61.0%)**	53 (71.6%)	27 (46.6%)	60.7%
Euclidean/squared inverse weights	20 (48.8%)	46 (62.2%)	26 (44.8%)	53.2%
Standardized Euclidean/equal weights	22 (53.7%)	54 (73.0%)	26 (44.8%)	59.0%
Standardized Euclidean/inverse weights	**25 (61.0%)**	53 (71.6%)	27 (46.6%)	60.7%
Standardized Euclidean/squared inverse weights	20 (48.8%)	46 (62.2%)	26 (44.8%)	53.2%
Spearman/equal weights	15 (36.6%)	59 (79.7%)	19 (32.8%)	53.8%
Spearman/inverse weights	17 (41.5%)	61 (82.4%)	19 (32.8%)	56.1%
Spearman/squared inverse weights	17 (41.5%)	56 (75.7%)	17 (29.3%)	52.0%
